# Paralyses. Central, Bulbar and Spinal

**Published:** 1887-02

**Authors:** 


### BIBLIOGRAPHY.

Paralyses, Central, Bulbar and Spinal, a manual of Diagnosis for students and practitioners, by H. Carlton Bastian, M. A. M. D. F. R. S. Fellow of the Royal College of Physicians, Examiner in Medicine at the Royal College of Physicians, Professor of Clinical Medicine and Pathological Anatomy in University College, London, Physician to University College Hospital, Etc.

With numerous illustrations, New York, 1886, D. Appleton & Co. 1, 3 and 5 Bond street.

This work may be said to be elementary, in its nature; it deals with the a b c's of the subject—as it were,—which is all very well, and goes further. It is a good work on diagnosis,—it tells how to recognize certain dseases by symptoms, to trace effect to cause; but leaves the reader to determine on a course of treatment for himself, or to hunt up other authority. Perhaps it is the authors intention to follow it by a book giving the treatment; without which his book is incomplete. The author however, has accomplished much, in bringing together, in this form most of the important advances made in neuro pathology within the past decade:

